# Effect of Directional Solidification on Microstructural Evolution and Properties of GH3625 Alloy

**DOI:** 10.3390/ma19071442

**Published:** 2026-04-03

**Authors:** Yanqin Zhang, Zhi Jia, Yafei Liu

**Affiliations:** 1 State Key Laboratory of Advanced Processing and Recycling of Nonferrous Metals, Lanzhou University of Technology, Lanzhou 730050, China; 19029339565@163.com (Y.Z.); 15137280429@163.com (Y.L.); 2 School of Material Science and Engineering, Lanzhou University of Technology, Lanzhou 730050, China

**Keywords:** directional solidification, solid–liquid interface, microstructure refinement, Laves phase

## Abstract

Nickel-based superalloy GH3625 is widely used in extreme environments due to its exceptional high-temperature strength and corrosion resistance; however, optimizing its comprehensive performance through precise microstructural control remains a critical challenge. In this study, the effect of withdrawal rate (10–200 μm/s) on the microstructural evolution, mechanical properties, and corrosion resistance of GH3625 alloy was investigated using a liquid-metal-cooled directional solidification system. The microstructural characteristics, elemental segregation, and phase distributions were systematically analyzed via OM, SEM, and EDS, followed by uniaxial tensile and electrochemical polarization tests. The results show that with increasing withdrawal rate, the solid–liquid interface morphology evolves from cellular to cellular-dendritic and finally to fully dendritic. Correspondingly, the primary dendrite arm spacing decreases from 270.4 μm to 100.2 μm, and the secondary dendrite arm spacing decreases from 66.5 μm to 12.3 μm. The area fraction of the detrimental Laves phase first decreases and then increases, reaching a minimum at 100 μm/s. Correspondingly, the yield strength increases from 282 MPa to 409 MPa, and the corrosion resistance is optimized at 100 μm/s. The microstructure–property relationships are discussed based on second-phase strengthening theory and microstructural refinement. This study provides a theoretical basis and practical process windows for optimizing directional solidification parameters to achieve enhanced mechanical and corrosion performance in GH3625 alloy.

## 1. Introduction

GH3625 alloy is a solution-strengthened nickel-based superalloy widely used in aerospace, nuclear energy, and marine industries due to its excellent high-temperature creep resistance, oxidation resistance, and corrosion resistance [1,2]. With the development of these fields, higher requirements are placed on the comprehensive properties of GH3625 alloy. Recently, the exceptional corrosion resistance of the GH3625 alloy has expanded its potential into novel biomedical applications, including metallic bone-mimicking scaffolds [3,4,5]. However, a critical gap remains: a quantitative understanding of how extreme directional solidification parameters dictate its precise microsegregation and micro-galvanic corrosion mechanisms is still lacking. Therefore, this study aims to elucidate the intrinsic relationship between withdrawal rates, microstructural refinement, and macroscopic properties.

Extensive studies have demonstrated that withdrawal rate is a critical parameter in directional solidification, governing the solid–liquid interface morphology, dendritic refinement, and segregation behavior [6,7,8]. For nickel-based superalloys containing segregation-prone elements such as Nb and Mo, the withdrawal rate directly influences solute redistribution and the formation of secondary phases [9,10,11]. Taken together, these findings confirm that by controlling the withdrawal rate, the microstructure and properties of nickel-based superalloys can be effectively modified and optimized.

However, most studies on directional solidification technology have focused on other grades of nickel-based superalloys, while a systematic investigation of the microstructural evolution and properties of GH3625 alloy under directional solidification conditions remains lacking. Therefore, this study focuses on the effect of withdrawal rate on the microstructure and properties of GH3625 alloy. Optical microscopy (OM), scanning electron microscopy (SEM), and energy-dispersive spectroscopy (EDS) are employed to systematically analyze the dendrite morphology, phase composition, elemental segregation behavior, and secondary phase precipitation characteristics at different withdrawal rates. Combined with mechanical property and corrosion resistance testing, the influence of withdrawal rate on microstructural evolution and material performance is elucidated, aiming to establish the intrinsic relationship between process parameters, microstructure, and properties.

## 2. Materials and Methods

### 2.1. Directional Solidification Experiment

The chemical composition of the GH3625 alloy ingot used in this investigation is listed in Table 1. First, cylindrical specimens with a diameter of 7 mm and a length of 110 mm were prepared from the alloy ingot by wire-cut electrical discharge machining (EDM). The specimens were then subjected to surface treatment, including cleaning and grinding, to remove surface scale and oxide residues.

**Figure 1:**
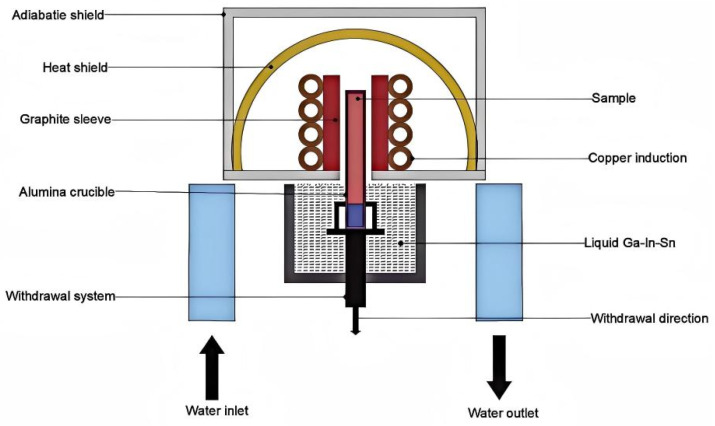
Directional solidification system diagram.

A liquid-metal-cooled (LMC) directional solidification system (schematic depicted in Figure 1) was used in the experiment. The specimen was placed inside the furnace in an Al_2_O_3_ crucible, and the gas supply and inlet valves were closed one after the other. The chamber was then evacuated by opening the fore-vacuum and main vacuum valves. The diffusion pump and fore-vacuum valve were turned on for a 40 min preheating period once the vacuum level fell below 2 × 10^−1^ Pa. Argon was added as a protective gas when the system vacuum reached 2 × 10^−2^ Pa. The following parameters were set for the process: growth length of 60 mm; heating temperature of 1400 °C; and withdrawal rates of 10, 20, 50, 100, and 200 μm/s. The withdrawal rates (10, 20, 50, 100, and 200 μm/s) were selected based on preliminary experiments covering the typical range for directional solidification of nickel-based superalloys. The upper limit was determined by the cooling capacity of the liquid metal coolant (Ga-In-Sn alloy). The directional solidification procedure was started after 30 min of holding. To preserve the solid–liquid interface morphology, the specimen was quenched into liquid Ga-In-Sn alloy immediately after solidification. Each experimental condition was repeated three times to ensure reproducibility. After the apparatus cooled to room temperature, the heating power source was switched off and the sample was taken out.

### 2.2. Sample Characterization

Along with longitudinal and cross-sectional sections taken from the steady-state growth region, a 20 mm long section containing the solid–liquid interface along the sample’s longitudinal axis was removed. After polishing and grinding, an oxalic acid solution (distilled water: oxalic acid = 9:1 *v*/*v*) was used for microstructural etching in a fume hood. The sample was immersed for 60–80 s, then promptly rinsed with anhydrous ethanol and allowed to air-dry.

An optical microscope (OM) (Carl Zeiss AG, Jena, Germany) was mainly used to observe the morphology of the solid–liquid interface. A laser scanning confocal microscope (LSCM, model LSM-800, Carl Zeiss Microscopy GMBH, Jena, Germany) and a scanning electron microscope (SEM, model Quanta FEG-450, FEI Company, Hillsboro, OR, USA) were used to examine microstructural evolution characteristics.

### 2.3. Tensile Properties Test

**Figure 2:**
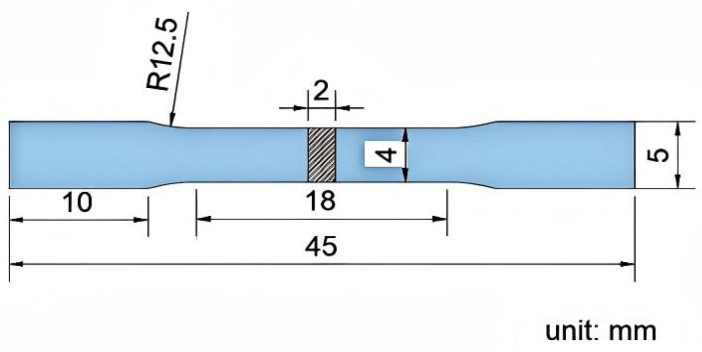
Dimensions of Tensile Specimen.

To ensure statistical reliability, three specimens were tested for each withdrawal rate. Repeated tests were performed to reduce the influence of random errors, material heterogeneity, and experimental uncertainties. Tensile specimens were sectioned from the central region of the directionally solidified bar (indicated by the reference lines in Figure 2) to ensure sampling from the steady-state growth region. To minimize errors introduced by wire cutting and subsequent fine grinding, a 0.5 mm allowance was reserved on each side of the cutting line.

Surface oxide layers were removed by grinding with silicon carbide sandpaper ranging from 400# to 2000# grit. Mechanical property tests were performed on a WDW-100D universal testing machine with a maximum load capacity of 100 kN, using a constant tensile rate of 0.5 mm/min. Strain was monitored with an extensometer having a gauge length of 10 mm. The analysis of plastic deformation behavior and stress–strain relationships in this work is derived from data collected within the extensometer’s valid measurement range. Tensile strength was determined from the maximum load (F_m_) recorded by the machine’s load cell and the specimen’s initial cross-sectional area (A_0_). The extensometer’s full-range strain measurement was not used in this calculation [12].

### 2.4. Electrochemical Corrosion Performance Testing

A 7 mm × 7 mm test specimen was cut from the longitudinal cross-section of the steady-state growth region at each withdrawal rate. The specimen was encapsulated in epoxy resin, exposing a working surface area of 1 cm^2^ after grinding with SiC abrasive papers (ranging from 400# to 2000# grit). Following resin curing, the exposed surface was sequentially ground using 2000# to 3000# grit papers and polished with diamond paste. The specimen was then rinsed with ethanol and deionized water.

Electrochemical tests were performed on a CHI660D workstation using a three-electrode configuration: the specimen as the working electrode, a platinum plate as the counter electrode, and a saturated calomel electrode (SCE) as the reference electrode. The electrolyte was a 3.5 wt.% NaCl aqueous solution. Dynamic potentiodynamic polarization tests were conducted at room temperature with a potential range of −2 V to +1.5 V (vs. SCE) and a scan rate of 1 mV/s. Electrochemical impedance spectroscopy (EIS) measurements were performed at the open-circuit potential with an excitation signal amplitude of 10 mV over a frequency range of 10^−2^ Hz to 10^5^ Hz. All data were collected and processed using Origin 2021 software.

## 3. Results and Discussion

### 3.1. GH3625 Alloy Original Microstructure

**Figure 3:**
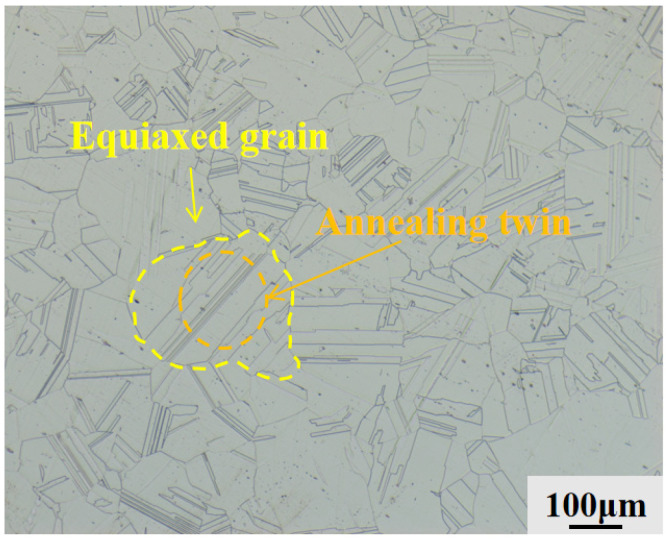
Optical microscopy (OM) image of the as-cast GH3625 alloy.

Figure 3 shows an optical microscopy (OM) image of the as-cast microstructure of the GH3625 alloy. Analysis of the OM image indicates that the GH3625 alloy exhibits typical fully recrystallized microstructural characteristics in the as-cast condition. The microstructure primarily consists of equiaxed grains, which contain numerous annealing twins. Most of these twins extend across the entire grain, although some terminate within the grain interior. This microstructural feature suggests that the GH3625 alloy underwent solution heat treatment at a temperature of at least 1030 °C.

**Figure 4:**
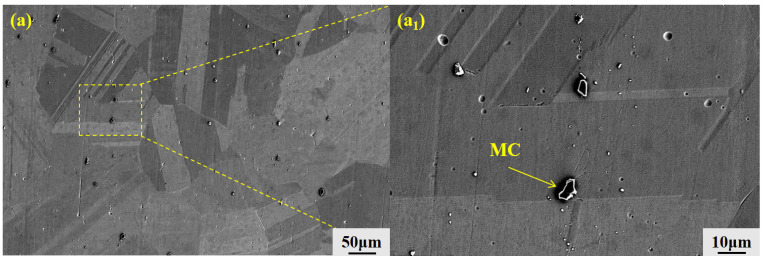
(**a**) Scanning electron microscopy (SEM) image of the as-cast microstructure of GH3625 alloy; (**a_1_**) localized magnification of a.

The presence and distribution of distinct secondary phases within the as-cast microstructure of the alloy are revealed by scanning electron microscopy (SEM) images and a corresponding high-magnification view in Figure 4a,a_1_. Further energy-dispersive spectroscopy (EDS) analysis confirms that the as-cast GH3625 alloy contains a significant amount of blocky MC-type carbides. While a few of these carbides are located near grain boundaries, the majority are distributed within the grain interiors.

### 3.2. Microstructure of GH3625 Alloy After Directional Solidification

#### 3.2.1. Evolution and Mechanisms of Solid–Liquid Interface Morphology

**Figure 5:**
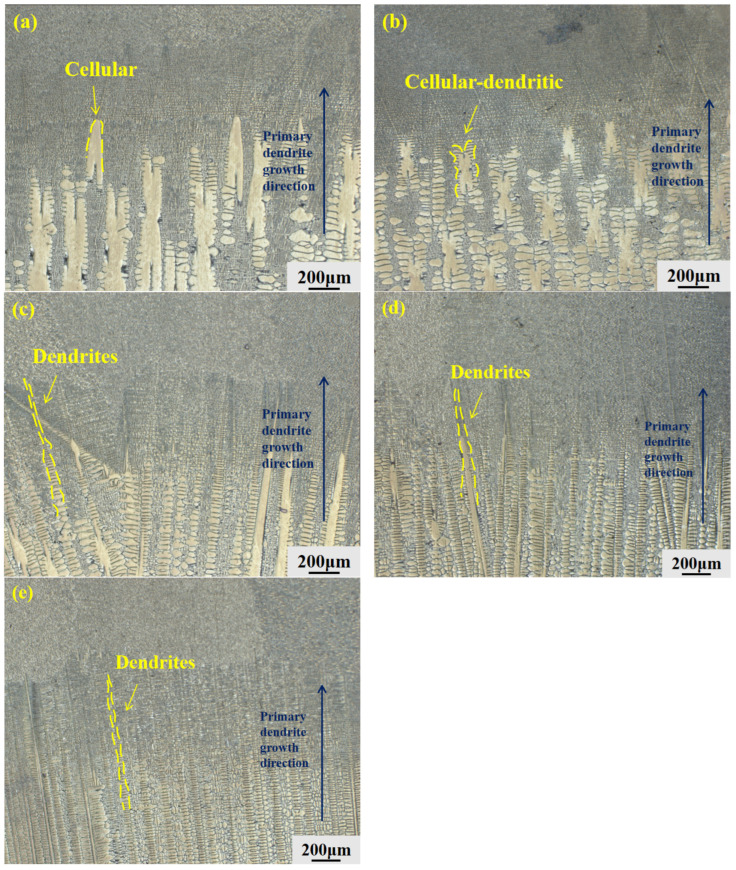
S/L interface morphology of GH3625 alloy at different withdrawal rates: (**a**) 10 μm/s, cellular structure; (**b**) 20 μm/s, cellular-dendritic transition; (**c**) 50 μm/s, dendritic structure; (**d**) 100 μm/s, refined dendrites; (**e**) 200 μm/s, disordered dendrites. Dark blue arrows indicate the primary dendrite growth direction.

**Figure 6:**
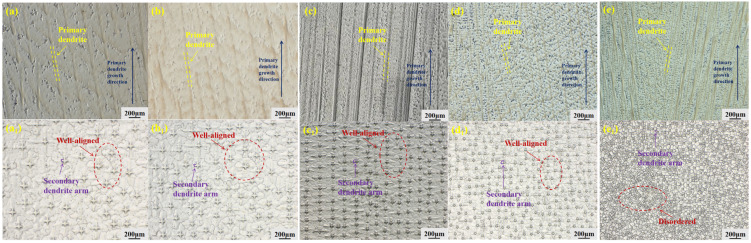
Longitudinal cross-sections (**a**–**e**) and transverse cross-sections (**a_1_**–**e_1_**) of dendritic structures in GH3625 alloy at different withdrawal rates: (**a**,**a_1_**) 10 μm/s, (**b**,**b_1_**) 20 μm/s, (**c**,**c_1_**) 50 μm/s, (**d**,**d_1_**) 100 μm/s, (**e**,**e_1_**) 200 μm/s.

Following directional solidification at various withdrawal rates, the microstructure of the GH3625 alloy underwent significant changes compared to its as-cast condition. Unlike the randomly oriented equiaxed grains observed in the as-cast alloy (Figure 3) [13], the microstructure is now entirely composed of columnar dendrites preferentially aligned along the direction of heat flow. Systematic examination of the solid–liquid (S-L) interface morphology presented in Figure 5, together with the corresponding microstructures in the steady-state growth region shown in Figure 6, reveals a clear morphological evolution as the withdrawal rate increases from 10 μm/s to 200 μm/s. The interface morphology evolves progressively from cellular to cellular-dendritic and finally to fully dendritic structures.

This morphological evolution is primarily governed by constitutional undercooling during directional solidification. According to fundamental solidification theory, the microstructural evolution is controlled by the temperature gradient (G) ahead of the solid–liquid interface and the withdrawal rate v. In the LMC process, the liquid metal coolant provides an extremely high and relatively constant temperature gradient (G). Consequently, the withdrawal rate (v) becomes the dominant dynamic parameter. An increase in v leads to a decrease in the G/v ratio, which increases the constitutional undercooling ahead of the advancing interface, thus driving the transition from cellular to dendritic morphologies. This evolution is consistent with the Mullins–Sekerka interface stability theory, which predicts that when the G/R ratio falls below a critical value (where R is the withdrawal rate v), the planar interface becomes unstable and evolves into cellular and dendritic structures [14]. Simultaneously, the cooling rate increases with the withdrawal rate, directly resulting in the significant refinement of the dendritic structures. The experimentally observed transition ranges—10–20 μm/s for the cellular to cellular-dendritic transition and 20–50 μm/s for the cellular-dendritic to fully dendritic transition—are consistent with the theoretical predictions of the Mullins–Sekerka interface stability theory, where the critical transition occurs when the G/v ratio falls below a threshold value [15].

The microstructural features within the steady-state growth region of the GH3625 alloy at different withdrawal rates are presented in Figure 6. The longitudinal section exhibits a typical dendritic structure, while the transverse section reveals a distinct “cross-shaped” morphology. At withdrawal rates ranging from 10 to 100 μm/s, the dendrites display an orderly arrangement with well-defined four-fold symmetry. In contrast, a substantial suppression of dendritic growth at a withdrawal rate of 200 μm/s leads to a disordered dendritic arrangement.

**Figure 7:**
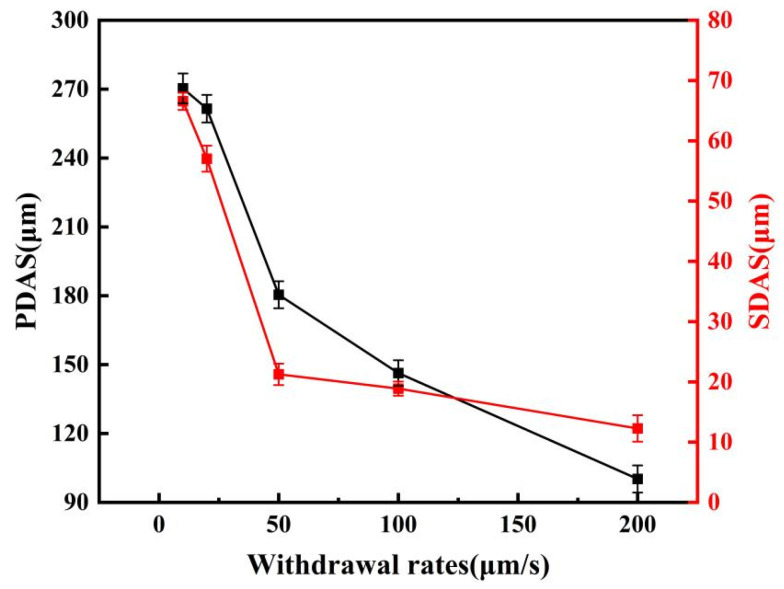
The primary and secondary dendritic arm spacings (PDAS and SDAS) of the alloy as a function of withdrawal rate. (n_1_ = 10 fields of view for λ_1_, n_2_ = 50 secondary dendrite arms for λ_2_).

From a microstructural perspective, the dendritic structure undergoes progressive refinement as the withdrawal rate increases within the investigated range of 10–200 μm/s. This evolution is clearly illustrated by the microstructural morphologies presented in Figure 5 and Figure 6. To quantitatively assess the degree of dendritic refinement, Dendrite arm spacings were measured using Image-Pro Plus 6.0 software. For primary dendrite arm spacing (λ_1_), the area method was applied on at least 10 fields of view per specimen at a magnification of 100×, selecting clearly defined primary trunks within the steady-state growth region. For secondary dendrite arm spacing (λ_2_), the line intercept method was used, measuring more than 50 secondary arms per specimen at a magnification of 500×; only well-developed arms with clear boundaries were included. All measurements were performed on specimens from three repeated experiments. The results are presented as mean ± standard deviation. Within the steady-state growth region, the primary dendritic arm spacing (λ_1_) decreased from 270.4 μm to 100.2 μm. The secondary dendrites, which reflect local microstructural details, were quantified using the line-intercept method. The results indicate that the secondary dendritic arm spacing (λ_2_) decreased from 66.5 μm to 12.3 μm, as show in Figure 7.

#### 3.2.2. Dendrite Arm Spacing Analysis

According to solidification theory, the primary dendrite arm spacing λ_1_ in the directionally solidified state follows a power-law relationship with the withdrawal rate v, which can be described by the Kurz–Fisher model [16]. Regression analysis of the measured λ_1_ data yields the following fitting equation:

(1) $$\lambda_{1}={510.2\cdot v}^{-0.43}(R^{2}=0.98)$$

The fitted exponent n = 0.43 (Equation (1)) falls within the theoretical range of 0.25–0.5. Notably, this value is closer to the upper bound of the theoretical range, indicating that the primary dendrite arm spacing is relatively sensitive to changes in withdrawal rate under the present solidification conditions.

The secondary dendrite arm spacing λ_2_ is closely related to the local solidification time during the solidification process and can theoretically also be described by a power-law function of the withdrawal rate. Since no unified theoretical model is currently applicable to all alloy systems, an empirical formula was obtained by power-law fitting of the experimentally measured secondary dendrite arm spacing data:

(2) $$\lambda_{2}={215.3\cdot v}^{-0.58}(R^{2}=0.96)$$

Equation (2) quantitatively describes the variation in λ_2_ with withdrawal rate. Together with the primary dendrite refinement, this secondary dendrite refinement contributes to the overall microstructural refinement that influences the mechanical properties and corrosion resistance discussed in Section 4. The observed refinement trend is valid only within the investigated range of 10–200 μm/s, as extrapolation beyond this range may not be valid due to potential changes in solidification mechanisms (e.g., absolute stability at extremely high growth rates or microstructural coarsening under very low growth rates).

#### 3.2.3. Analysis of Dendrite Growth Mechanism

Combining the morphological observations with the quantitative measurements, and considering the solid–liquid interface evolution shown in Figure 5, it can be inferred that at high withdrawal rates, the rate of heat extraction exceeds the diffusion capability of solute atoms. This imbalance in heat and mass transfer affects the final microstructure in two aspects. On one hand, the rapidly extracted heat flux forces the dendrites to increase their heat dissipation area through frequent branching to accommodate the higher cooling rate, which directly leads to the refinement of both primary (λ_1_) and secondary (λ_2_) dendrite arm spacings. On the other hand, when the withdrawal rate increases to 200 μm/s, the excessively rapid solidification front prevents adjacent dendrites from adjusting their growth directions in time, causing their growth trajectories to intersect and ultimately become unstable due to strong mechanical obstruction. This manifests macroscopically as disordered dendrite arrangement and intensified competitive growth.

#### 3.2.4. Competitive Growth and Segregation Behavior of Equiaxed Grains in the Mushy Zone

**Figure 8:**
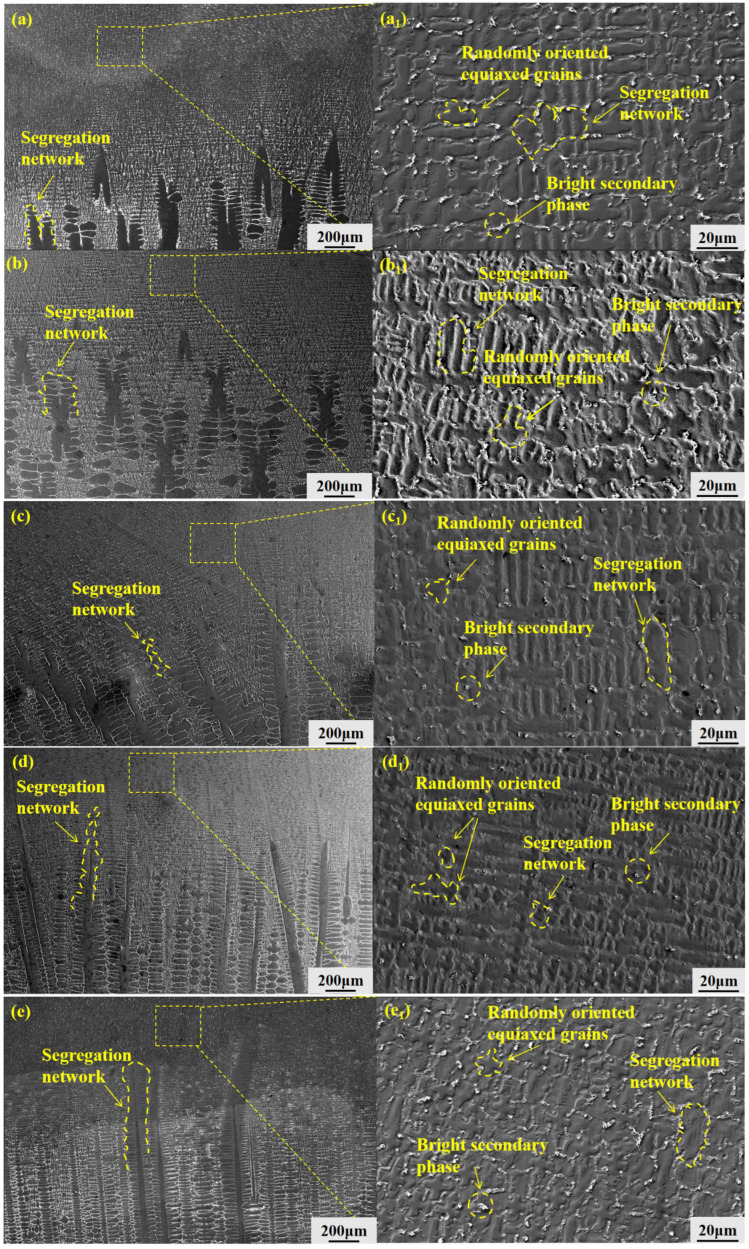
S-L interface and smeared zone morphology of GH3625 alloy at different drawing rates (**a**–**e**) and corresponding magnified views (**a_1_**–**e_1_**): (**a**,**a_1_**) 10 μm/s; (**b**,**b_1_**) 20 μm/s; (**c**,**c_1_**) 50 μm/s; (**d**,**d_1_**) 100 µm/s; (**e**,**e_1_**) 200 µm/s.

To investigate the microstructural evolution of GH3625 alloy during directional solidification, SEM analysis was conducted on samples solidified at different withdrawal rates (10–200 μm/s). As shown in Figure 8, directional solidification not only optimizes the crystallographic orientation and refines the dendritic structure but also significantly influences the distribution of secondary phases and microsegregation behavior.

A closer examination of the mushy zone reveals that randomly oriented equiaxed grains coexist at all withdrawal rates, with equiaxed grains frequently located ahead of the columnar dendrite tips. Bright secondary phases are preferentially precipitated along the equiaxed grain boundaries (as clearly observed in the transition zones of Figure 8a–e). This spatial arrangement indicates a competitive growth mechanism between columnar dendrites and equiaxed grains during directional solidification. Further EDS analysis reveals that the equiaxed grain boundaries are enriched in Nb, Mo, and Ti, acting as fast diffusion channels and preferential segregation sites. This leads to the formation of a continuous segregation network in the interdendritic regions, which connects the equiaxed grains with the surrounding columnar dendrites. This segregation behavior is consistent with the Scheil-type solute redistribution model [17].

The equiaxed grain boundaries act as fast diffusion channels for solute elements such as Nb, Mo, and Ti. When multiple equiaxed grains are interconnected, their grain boundaries form a continuous network that provides preferential paths for solute diffusion and microcrack propagation. This network is more pronounced at lower withdrawal rates due to the larger equiaxed grain size and more extensive interconnections. At higher withdrawal rates, the equiaxed grain region is compressed, reducing the connectivity of this network and thereby mitigating its detrimental effects. This dual heterogeneity in both composition and structure not only deteriorates microstructural uniformity but also renders these grain boundaries susceptible to microcrack initiation and propagation. However, the morphology and distribution of these heterogeneous features vary significantly with the withdrawal rate. At 10 μm/s (Figure 8a,a_1_), the dendrites are coarse, and the equiaxed grain regions are relatively extensive, occupying a large proportion of the microstructure and interweaving with the columnar dendrites. The bright secondary phases are densely distributed along the grain boundaries, forming large-scale segregation areas. As the withdrawal rate increases to 20–50 μm/s (Figure 8b,c,b_1_,c_1_), the dendrites begin to refine, and the equiaxed grain regions are gradually compressed, with their proportion in the microstructure decreasing accordingly. Columnar dendrites progressively become the dominant structural feature. The distribution range and density of the bright secondary phases are reduced compared to those at 10 μm/s, although they remain distributed along the interdendritic channels. At a withdrawal rate of 100 μm/s (Figure 8d,d_1_), the dendrites are significantly refined, and the size and extent of the equiaxed grain regions are further reduced. The bright secondary phases become considerably sparser, with their density markedly lower than that observed at 50 μm/s and below. When the withdrawal rate is further increased to 200 μm/s (Figure 8e,e_1_), despite further dendritic refinement, the bright secondary phases become coarse again, exhibiting significantly larger precipitated particles.

#### 3.2.5. Control of Solute Element Distribution and Segregation Behavior

**Figure 9:**
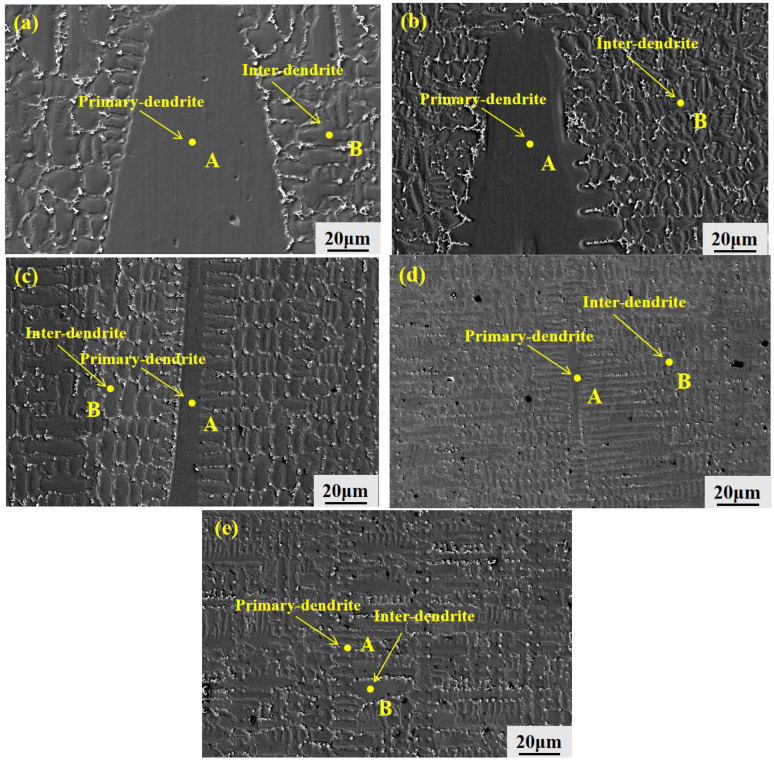
Dendritic morphology of GH3625 alloy at different withdrawal rates: (**a**) 10 μm/s; (**b**) 20 μm/s; (**c**) 50 μm/s; (**d**) 100 μm/s; (**e**) 200 μm/s; (A) primary dendrite arm; (B) interdendritic region.

To quantitatively evaluate the dendritic segregation behavior of the alloy, the segregation coefficient (k) was used to characterize the degree of segregation for each element. The segregation coefficient is defined as the ratio of the average concentration in the dendrite core (C_dendrite_) to that in the interdendritic region (C_interdendritic_), i.e., k = C_dendrite_/C_interdendritic_ [18]. When k > 1, the element is enriched in the interdendritic regions, indicating positive segregation; when k < 1, the element is enriched in the dendrite cores, indicating negative segregation. As indicated in Figure 9, the dendrite core (A) and interdendritic region (B) were clearly distinguishable. For each withdrawal rate, at least five pairs of corresponding positions in the dendrite cores and interdendritic regions were selected for EDS point analysis, and the average values were used to calculate the segregation coefficient.

**Figure 10:**
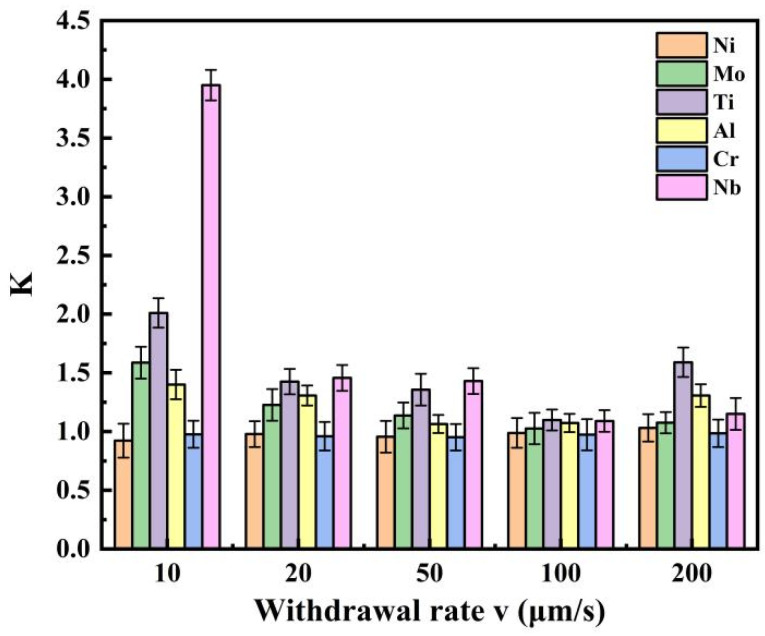
Variation in segregation coefficients of major elements in GH3625 alloy at different withdrawal rates. Each data point is based on five EDS measurements and presented as mean ± standard deviation.

Based on the above calculation method for dendritic segregation, a quantitative analysis of the segregation behavior of each element was conducted. As shown in Figure 10, the segregation degree of each element in the GH3625 alloy exhibits different characteristics with varying withdrawal rates. From an overall perspective, Nb exhibits the most significant segregation behavior, with its segregation coefficient remaining at a relatively high level at all withdrawal rates, followed by Al, Ti, and Mo, while the segregation coefficients of Cr and Ni are relatively low. In terms of distribution, Nb, Mo, Ti, and Al are enriched in the interdendritic regions, exhibiting positive segregation, whereas Ni and Cr are enriched in the dendrite cores, exhibiting negative segregation.

Further analysis focuses on the segregation behavior of Nb and Mo, as they are the key alloying elements governing the formation of strengthening phases and the overall properties of GH3625. The segregation coefficients of Nb and Mo decrease and then increase as the withdrawal rate increases. As shown in Table 2, the segregation ratio of Nb decreases from 4.05 at 10 μm/s to 1.07 at 100 μm/s, and then increases to 1.14 at 200 μm/s. Similarly, as shown in Table 3, the segregation ratio of Mo decreases from 1.60 at 10 μm/s to 1.001 at 100 μm/s, approaching equilibrium, and then increases to 1.014 at 200 μm/s. Therefore, the withdrawal rate has a significant regulatory effect on the segregation behavior of Nb and Mo, and both elements exhibit the lowest degree of segregation at 100 μm/s.

The solute redistribution behavior during solidification can be described by the Scheil equation [17]:

$$C_{s}={kC_{0}(1-f_{s})}^{k-1}$$

where C_s_ is the solute concentration in the solid phase, C_0_ is the initial solute concentration, k is the equilibrium partition coefficient, and f_s_ is the solid fraction. This model describes the solute partitioning behavior at the solid–liquid interface and can be used to understand the segregation evolution under different solidification rates.

In the present study, at a low withdrawal rate of 10 μm/s, the segregation coefficient of Nb reaches as high as 4.05, approaching the extreme segregation case predicted by the Scheil model. As the withdrawal rate increases to 20–100 μm/s, solute diffusion becomes inhibited, and the degree of segregation decreases. The Nb segregation coefficient drops to 1.07–1.45, which is significantly lower than the Scheil model prediction of approximately 1.5–2.0 under similar solidification conditions (assuming complete liquid mixing and no solid-state diffusion). This indicates that the “freezing” effect under diffusion-limited conditions suppresses complete solute redistribution, thereby promoting compositional homogenization to some extent.

At a withdrawal rate of 200 μm/s, the solidification rate becomes extremely high. Under such conditions, solute atoms do not have sufficient time to diffuse away from the advancing solid–liquid interface and are instead directly incorporated into the growing solid—a phenomenon known as solute trapping. As a result, Nb and Mo atoms are retained in the solid phase rather than being redistributed to the interdendritic regions, leading to local solute enrichment at the interface front. This causes the segregation coefficient of Nb to increase from 1.07 at 100 μm/s to 1.14 at 200 μm/s. This suggests that, beyond a critical withdrawal rate, the beneficial effect of dendrite refinement on reducing segregation may be partially offset by the enhanced solute trapping effect.

#### 3.2.6. Identification of Secondary Phases

**Figure 11:**
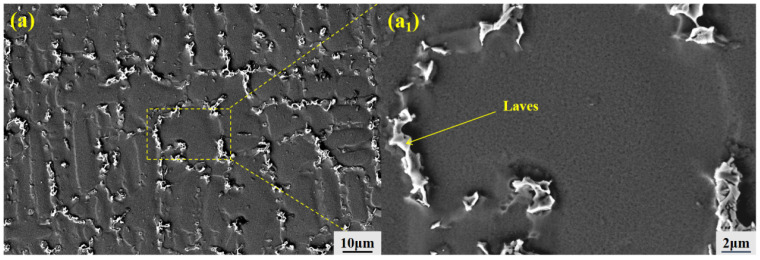
Laves phase morphology in directionally solidified GH3625 alloy at a withdrawal rate of 10 μm/s: (**a**) BSE image showing interdendritic Laves phase; (**a_1_**) higher-magnification view of the blocky phase.

**Figure 12:**
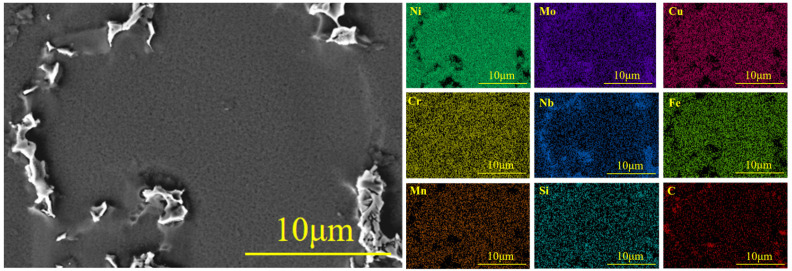
EDS Element Distribution Map of GH3625 Alloy with Directional Solidification: Backscattered electron image showing interdendritic precipitates.

As shown in Figure 11, large irregular second phases are clearly visible at the dendrite boundaries. EDS compositional analysis in Table 4 indicates that this phase contains relatively high amounts of Nb and Mo, while Ni and Cr contents are lower. Elemental mapping analysis in Figure 12 reveals that this precipitation phase precisely corresponds to the Nb- and Mo-enriched regions. Additionally, A dendrite-core versus interdendrite Ni-Cr-rich versus Nb-Mo-rich elemental partitioning is observed. Weak carbon (C) signals are also locally detectable, suggesting the possible presence of minor MC-type carbides. The aforementioned morphology, composition, and elemental distribution characteristics conform to the classical morphology and composition criteria of the Laves phase [(Ni,Cr,Fe)_2_(Nb,Mo)], consistent with findings from similar alloy studies [19,20].

Compared to the as-cast GH3625 alloy, the type of secondary phase in the directionally solidified specimen significantly changes from MC-type carbides to Laves phases. This transition is primarily attributed to the pronounced microsegregation of elements such as Nb and Mo during the final stage of solidification, which promotes a peritectic reaction at lower temperatures: MC + γ (Mo-, Nb- rich) → γ (depleted) + Laves phase [21].

It should be noted that the Laves phase, being a brittle intermetallic compound, adversely affects the properties of nickel-based superalloys. Consequently, precise control of the solidification process during manufacturing is essential to either suppress its formation or achieve a fine and dispersed distribution [22,23].

#### 3.2.7. Laves-Phase Morphology Evolution and Area Fraction

**Figure 13:**
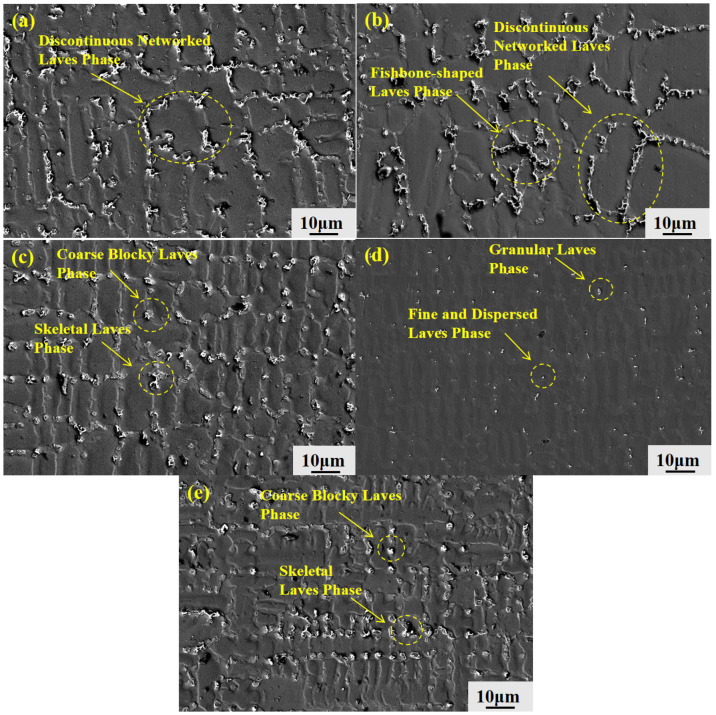
Morphology of Laves phases between dendrites at the S/L interface of GH3625 alloy at different withdrawal rates: (**a**) 10 μm/s; (**b**) 20 μm/s; (**c**) 50 μm/s; (**d**) 100 μm/s; (**e**) 200 μm/s.

The morphology of the Laves phase in the GH3625 alloy is significantly influenced by the withdrawal rate, as demonstrated by the data presented in Figure 13. The microstructure of the Laves phase undergoes continuous evolution as the withdrawal rate increases from 10 μm/s to 200 μm/s.

At a withdrawal rate of 10 μm/s, the Laves phase exhibited a bright, discontinuous network morphology. As the withdrawal rate increased to 20 μm/s, the majority of the Laves phase retained a discontinuous network pattern, while a minor fraction adopted a fishbone-like morphology. When the withdrawal rate was raised to 50 μm/s, the Laves phase transformed into coarse blocky and skeletal morphologies. A further increase in the withdrawal rate to 100 μm/s resulted in a fine, dispersed distribution of blocky and granular Laves-phase particles. However, at a withdrawal rate of 200 μm/s, the Laves phase reverted to coarse blocky and skeletal morphologies.

**Figure 14:**
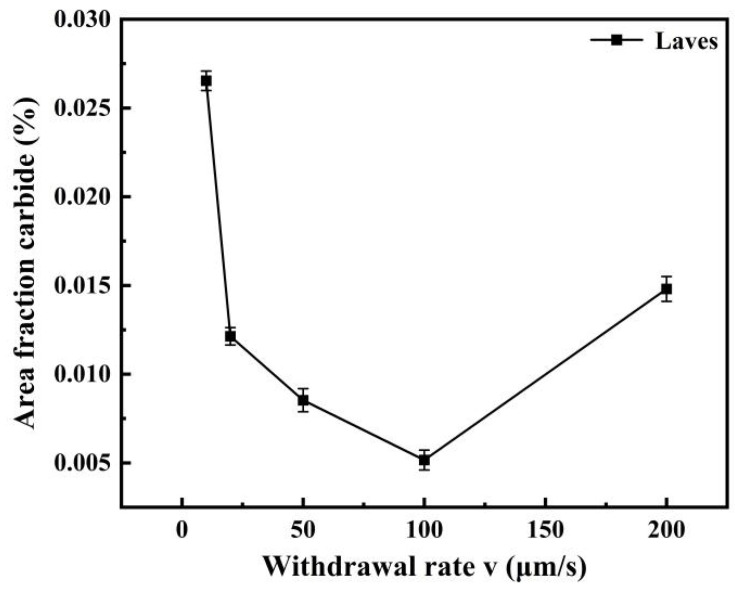
Area fraction of Laves phase in GH3625 alloy at different withdrawal rates. Data are presented as mean ± standard deviation (n = 5 fields of view).

Based on the observations presented in Figure 13, the area fraction of the Laves-phase interface was quantified using Image-Pro Plus 6.0 software. Five fields of view were measured for each withdrawal rate, and the results are presented as mean ± standard deviation. As shown in Figure 14, the area fraction of the Laves phase exhibited an initial decrease followed by an increase with rising withdrawal rate. Specifically, the value decreased from 2.68% at 10 μm/s to 0.56% at 100 μm/s, before increasing to 2.17% at 200 μm/s.

This variation in area fraction is consistent with the morphological evolution of the Laves phase. The morphological evolution of the Laves phase is governed by solute diffusion, dendrite refinement, and solidification undercooling, directly correlating with the segregation behavior discussed in Section 3.2.4 and Section 3.2.5. At 10 μm/s, slow solidification and coarse dendrites allow sufficient solute diffusion, leading to severe segregation and a discontinuous network Laves phase. As the withdrawal rate increases to 20–50 μm/s, dendrites refine and diffusion becomes limited, suppressing segregation and breaking the Laves phase into fishbone-like and blocky morphologies. At 100 μm/s, dendrites are significantly refined, and diffusion is largely suppressed, minimizing segregation and confining the Laves phase to a fine, dispersed distribution. At 200 μm/s, however, the mechanism shifts from diffusion-limited freezing to solute trapping. Despite further dendrite refinement, extreme local supersaturation from solute trapping drives rapid non-equilibrium growth along preferred crystallographic directions, while limited atomic diffusion prevents compact blocky formation, resulting in coarse blocky and skeletal morphologies.

### 3.3. Effect of Withdrawal Rate on the Correlation Between Microstructure, Mechanical Properties, and Fracture Mechanism

**Figure 15:**
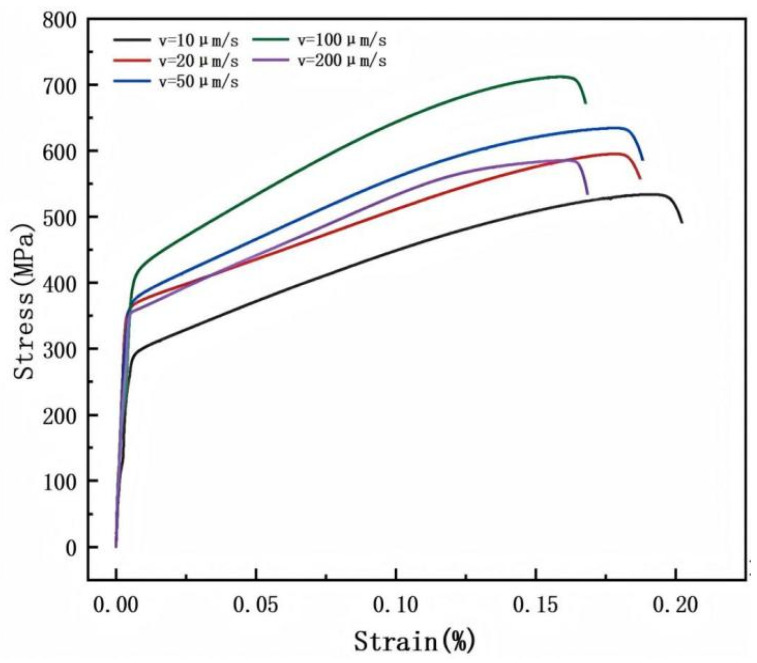
Tensile properties of GH3625 alloy at different withdrawal rates.

Figure 15 presents the tensile stress–strain curves of the GH3625 alloy at different withdrawal rates. As shown, when the withdrawal rate increases from 10 μm/s to 100 μm/s, the tensile strength of the alloy rises from 541 MPa to 712 MPa, and its yield strength increases from 282 MPa to 409 MPa. However, when the withdrawal rate is further increased to 200 μm/s, the tensile strength decreases to 586 MPa and the yield strength drops to 350 MPa. Three tensile specimens were tested for each withdrawal rate, and the results are presented as mean ± standard deviation. Representative stress–strain curves are shown.

**Figure 16:**
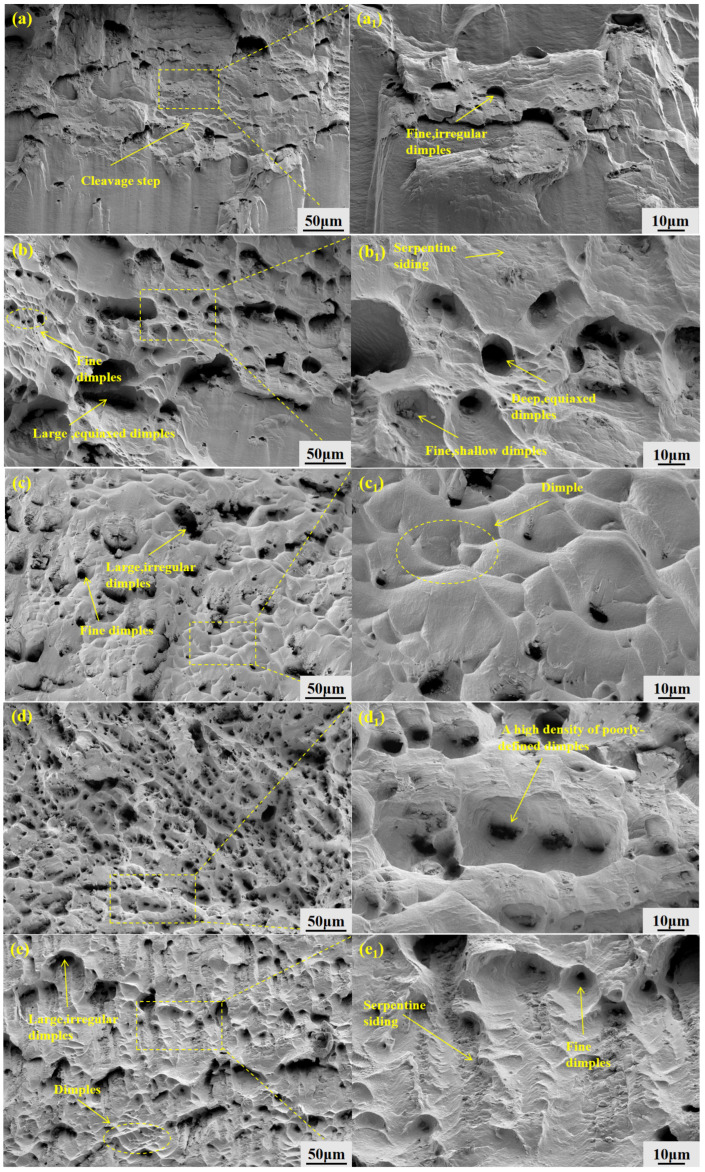
Fracture surface morphology of GH3625 alloy at different withdrawal rates (**a**,**b**) and corresponding magnified views (**a_1_**–**e_1_**): (**a**,**a_1_**) 10 μm/s; (**b**,**b_1_**) 20 μm/s; (**c**,**c_1_**) 50 μm/s; (**d**,**d_1_**) 100 µm/s; (**e**,**e_1_**) 200 µm/s.

Figure 16 presents the fracture morphologies of the GH3625 alloy at different withdrawal rates. At a withdrawal rate of 10 μm/s, the fracture surface exhibits numerous cleavage steps and irregular, shallow ductile dimples, indicative of a quasi-cleavage fracture mode, which suggests limited ductility of the material. Compared to the 10 μm/s condition, the fracture surface at a withdrawal rate of 20 μm/s contains a higher proportion of deep, equiaxed dimples. While still characteristic of quasi-cleavage fracture, these dimples reflect enhanced ductility. When the withdrawal rate is 50 μm/s, the fracture surface morphology is dominated by large, deep equiaxed dimples accompanied by occasional tearing ridges, indicating a transition towards a typical ductile fracture mechanism. At a withdrawal rate of 100 μm/s, As the rate continued to increase to 100 μm/s, the fracture surface retained ductile fracture characteristics but exhibited a high density of ductile dimples, indicating a reduction in material plasticity. The fracture surface at 200 μm/s exhibits a mixed-mode morphology: while deep equiaxed dimples predominate, features such as river patterns and some micro-cracks are also present. This results in a hybrid fracture mechanism that is primarily ductile with localized brittle components, ultimately manifesting as quasi-cleavage fracture.

The evolution of the fracture morphology is closely related to the morphological evolution of the Laves phase. To further investigate the relationship between the strength variation and the Laves phase morphology, the above strength data were compared with the statistical results of the Laves phase area fraction from Section 3.2.7 (Figure 13). To quantitatively evaluate the influence of the Laves phase on strength, the Laves phase area fraction was selected as a quantitative indicator, as it directly reflects the content and distribution density of the Laves phase and is easy to measure statistically. According to the second-phase strengthening theory, the contribution of the second-phase Laves phase to strength generally follows a power-law relationship σ ∝ f^m^, where f is the volume fraction of the second phase and m is a negative exponent [24]. A power-law fitting of the Laves phase area fraction (f) and yield strength (σ_y_) yields:

$$\sigma_{y}=319.5f^{-0.25}(R^{2}=0.99)$$

A power-law fitting of the Laves phase area fraction (f) and ultimate tensile strength (σ_UTS_) yields:

$$\sigma_{UTS}=487.2f^{-0.18}(R^{2}=0.94)$$

The fitting results indicate that the Laves phase area fraction exhibits a negative correlation with both yield strength and ultimate tensile strength, with a stronger correlation observed for yield strength. The fitted exponents m = −0.25 for yield strength and m = −0.18 for tensile strength are consistent with the Orowan strengthening mechanism, where finer and more dispersed Laves phases provide more effective obstacles to dislocation motion. Accordingly, as the Laves phase area fraction decreases from 2.68% at 10 μm/s to 0.56% at 100 μm/s, the yield strength increases from 282 MPa to 409 MPa. However, when the withdrawal rate reaches 200 μm/s, the Laves phase area fraction increases while the strength decreases. This may be attributed to the coarse, blocky Laves phase acting as local stress concentration sites, where the second-phase strengthening mechanism is offset by the stress concentration effect.

On the other hand, the evolution of the fracture morphology is also closely related to the morphological evolution of the equiaxed grain region with increasing withdrawal rate. At 10 μm/s, the equiaxed grain region is relatively extensive and interweaves with the columnar dendrites, forming a continuous mixed microstructure network that provides interconnected low-resistance paths for crack propagation. Cracks tend to propagate rapidly along these regions, resulting in a fracture surface characterized by cleavage steps, indicative of quasi-cleavage fracture. As the withdrawal rate increases to 20–50 μm/s, the equiaxed grain region is gradually compressed, its proportion in the microstructure decreases, and the continuity of the mixed microstructure is reduced. Crack propagation paths become more tortuous, requiring higher energy consumption, leading to the appearance of more deep dimples in the fracture surface and improved mechanical properties. When the withdrawal rate reaches 100 μm/s, the equiaxed grain region is further compressed, becoming small and confined. At this stage, the microstructure is dominated by refined columnar dendrites, and the equiaxed grain region can no longer form an interconnected network. Crack propagation cannot proceed along continuous paths and must instead bypass these compressed regions or renucleate, significantly increasing the energy required for fracture. Therefore, although the mixed microstructure still exists, its compressed morphology is insufficient to constitute a connected weak network. The fracture surface exhibits a high density of dimples, and both strength and plasticity reach their optimal values simultaneously. When the withdrawal rate increases to 200 μm/s, although the equiaxed grain region remains compressed, the coarse, blocky Laves phases become new stress concentration sites, which can directly initiate microcracks, leading to the reappearance of local brittle features such as river patterns in the fracture surface and a subsequent decrease in mechanical properties.

In conclusion, Among the withdrawal rates examined in this study, 100 μm/s yields the best overall properties. Under this condition, the detrimental effect of the mixed microstructure is effectively suppressed, the Laves phase is fine and dispersed, and the fracture surface exhibits a high-density dimple morphology, achieving typical ductile fracture. This provides an effective processing direction for optimizing the performance of the GH3625 alloy.

### 3.4. Analysis of Electrochemical Corrosion Behavior at Different Withdrawal Rates

#### 3.4.1. Potentiodynamic Polarization Test

**Figure 17:**
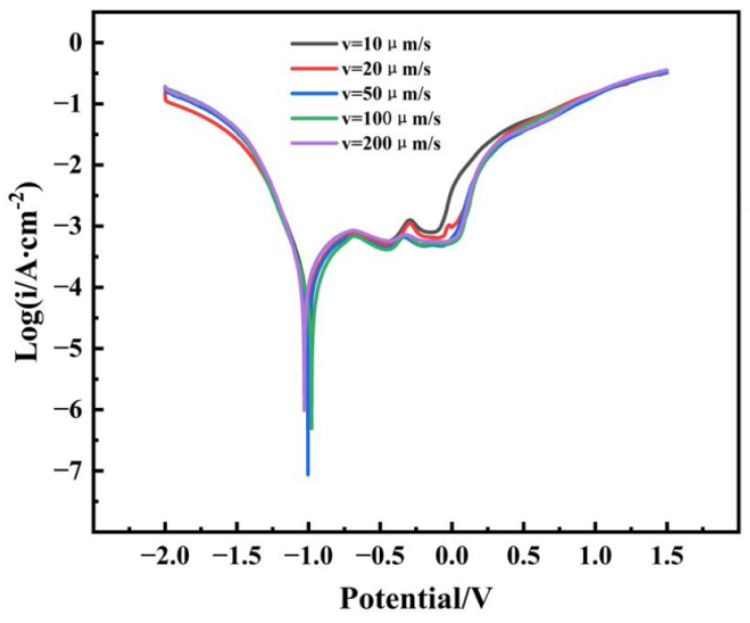
The potentiodynamic polarization tests were conducted in 3.5 wt.% NaCl solution for specimens solidified at withdrawal rates of 10, 20, 50, 100, and 200 μm/s. Three tests were performed for each condition, and representative curves are shown.

Each electrochemical test was repeated three times to ensure reproducibility. Representative polarization curves are shown, and electrochemical parameters (e.g., corrosion potential E_corr_, corrosion current density I_corr_ are presented as mean ± standard deviation. The electrochemical test results presented in Figure 17 demonstrate a clear correlation between the corrosion resistance of the directionally solidified GH3625 alloy and the withdrawal rate. Although the open-circuit potential (OCP) at a withdrawal rate of 10 μm/s is relatively high in Table 5, the pitting potential E_pit_ serves as the primary indicator for evaluating the alloy’s corrosion resistance [25]. The potentiodynamic polarization curves in Figure 17 reveal typical active dissolution and passivation regions for samples solidified at different withdrawal rates. The resistance to pitting corrosion follows the order: 100 μm/s > 50 μm/s > 200 μm/s > 20 μm/s > 10 μm/s (Table 6). Furthermore, the sample corresponding to a withdrawal rate of 100 μm/s exhibits the lowest passivation current density i_p_, indicating the lowest corrosion rate and the highest resistance to surface reactions. These findings suggest that both dendritic refinement and reduced microsegregation contribute to the enhancement of the material’s surface corrosion resistance. The improved corrosion resistance at 100 μm/s can be attributed primarily to the change in Laves phase distribution from a continuous network to a fine, dispersed morphology. This transformation isolates the micro-galvanic couples and prevents the formation of continuous corrosion paths, which is the dominant factor. Additionally, the refined dendritic structure reduces microsegregation, leading to a more uniform chemical composition and a more stable passive film, which further contribute to the enhanced corrosion resistance.

#### 3.4.2. Electrochemical Impedance Spectroscopy (EIS)

Electrochemical impedance spectroscopy (EIS) measurements were repeated three times for each condition to ensure reproducibility. Representative Nyquist and Bode plots are shown, and fitted electrochemical parameters (e.g., charge transfer resistance Rct) are presented as mean ± standard deviation.

**Figure 18:**
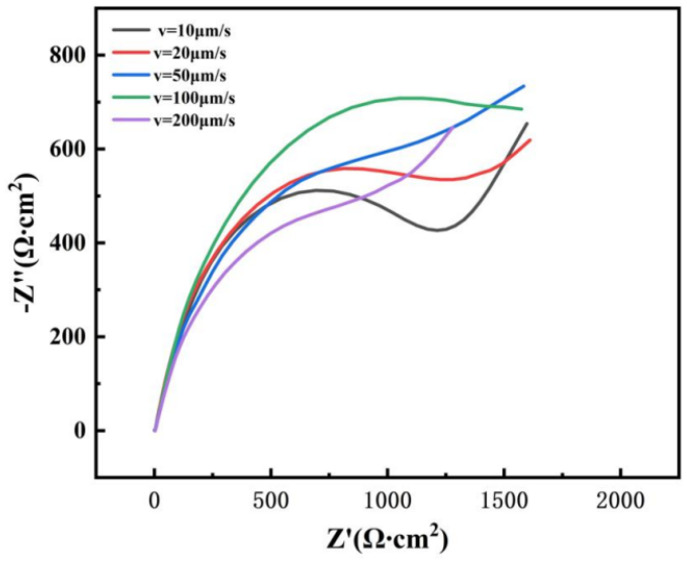
Nyquist plots of GH3625 alloy in 3.5 wt.% NaCl solution for different withdrawal rates (10, 20, 50, 100, 200 μm/s). Three tests were performed for each condition, and representative curves are shown.

According to the Nyquist plot presented in Figure 18, the specimen solidified at a withdrawal rate of 100 μm/s exhibits the largest capacitive-arc diameter, indicating the highest charge-transfer resistance among all samples. The ranking of the capacitive-arc diameters corresponds to the degree of pitting corrosion resistance.

**Figure 19:**
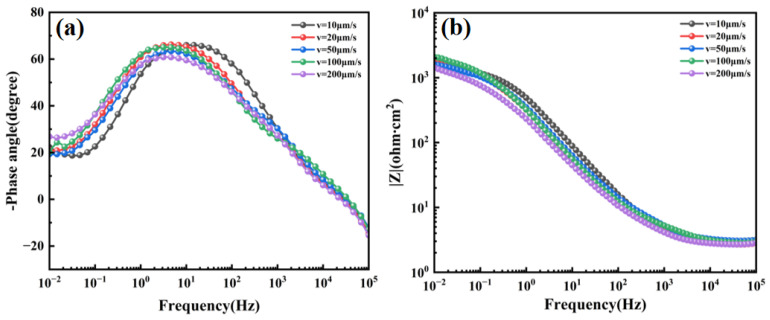
Bode plots of GH3625 alloy at different withdrawal rates in 3.5 wt.% NaCl solution: (**a**) Bode phase angle plot; (**b**) Bode magnitude plot. Three tests were performed for each condition, and representative curves are shown.

According to the Bode phase angle plot in Figure 19a, the sample solidified at a withdrawal rate of 100 μm/s exhibits the largest and widest phase-angle peak, indicating a uniform and stable interface. The state of the interface is governed by micro-electrochemical inhomogeneity: a uniform, fine distribution of secondary phases promotes an ideal capacitive interface, whereas coarse, segregated phases lead to multi-time-constant responses and reduced surface uniformity. The phase-angle peaks for samples at other withdrawal rates are either lower or exhibit broadening.

In electrochemical impedance spectroscopy, the low-frequency region (typically 0.01–0.1 Hz) primarily reflects the charge transfer resistance at the electrode/electrolyte interface, which is directly related to the corrosion resistance of the material. A higher impedance modulus |Z| in this region indicates a more stable passive film and greater resistance to charge transfer. When combined with the Bode magnitude plot analysis in Figure 19b, the impedance modulus |Z| at the characteristic frequency of 0.01 Hz is highest for the 100 μm/s sample and lowest for the 200 μm/s sample. These results suggest that the distribution and morphology of the Laves phase concurrently govern both the low-frequency impedance and the charge-transfer resistance. This can be explained by the micro-galvanic corrosion mechanism.

The Laves phase has a higher electrochemical potential than the γ matrix, creating micro-galvanic couples at the phase boundaries. When the Laves phase is continuously distributed (10 μm/s), these galvanic couples form a connected network, accelerating corrosion along the phase boundaries. In contrast, when the Laves phase is finely dispersed (100 μm/s), the galvanic effects are localized and less detrimental. At 200 μm/s, the coarse blocky Laves phase reintroduces significant galvanic coupling, leading to the formation of localized corrosion pits.

In conclusion, the GH3625 alloy demonstrates optimal corrosion resistance when a uniform microstructure is achieved at a withdrawal rate of 100 μm/s. Deviation from this process window results in a significant deterioration of performance.

## 4. Conclusions

(1) During directional solidification, the formation of segregated structures and the precipitation of Laves phases are governed by the following mechanism: elements such as Nb, Mo, and Ti are rejected by the initially solidified dendritic cores, leading to their enrichment in the interdendritic regions. Ultimately, this results in an interdendritic γ matrix enriched in Nb and Mo and a dendritic core γ matrix depleted in these elements. Subsequently, due to the enrichment of Nb and Mo in the interdendritic regions, the γ′ phase forms. Finally, when the concentrations of Nb and Mo reach saturation and cannot be fully dissolved in the γ matrix, they precipitate as Laves phases through eutectic/eutectoid reactions.
(2) The primary mechanism by which the withdrawal rate regulates the microstructure of the GH3625 alloy is through the control of solidification undercooling and solid–liquid interface stability, which subsequently influences the morphology of both dendrites and the Laves phase. As the withdrawal rate increases, the alloy’s structure progressively transitions from a cellular morphology to well-developed dendrites, accompanied by a continuous reduction in dendritic size (λ_1_: 270.4 μm → 100.2 μm; λ_2_: 66.5 μm → 12.3 μm). Correspondingly, the morphology of the Laves phase undergoes a phased evolution: at low withdrawal rates (10–20 μm/s), it appears as a discontinuous network or fishbone-like pattern; at 50 μm/s, it transforms into coarse blocky structures; at 100 μm/s, it refines into a fine, dispersed state; and at 200 μm/s, it coarsens again. The optimal condition, characterized by a refined microstructure and minimized segregation, is achieved at a withdrawal rate of 100 μm/s. At this rate, the dendrites are significantly refined while segregation is effectively suppressed, corresponding to the lowest measured area fraction of the Laves phase. Deviating from this optimal process window disrupt this balance: lower withdrawal rates produce coarse microstructures with pronounced segregation, while higher rates maintain refined dendrites but exacerbate microsegregation.
(3) The withdrawal rate directly influences the mechanical properties and fracture mechanisms ofthe GH3625 alloy by altering the morphology and distribution of Laves phases. At a withdrawal rate of 100 μm/s, the Laves phases exhibit a fine, dispersed morphology, which is conducive to a typical ductile fracture mode. Concurrently, both tensile strength and yield strength reach their maximum values. Conversely, when the withdrawal rate deviates from this optimal range (e.g., 10–50 μ m/s or 200 μ m/s), coarse Laves phases act as stress concentration sites, readily inducing quasi-cleavage fracture and consequently degrading the material’ s mechanical properties.
(4) Electrochemical corrosion test results demonstrate that the GH3625 alloy exhibits optimal corrosion resistance at a withdrawal rate of 100 μm/s. Under these process conditions, the alloy shows the highest pitting potential E_pit_, the largest capacitive-arc diameter, the lowest passivation current density i_p_, and the maximum impedance modulus in the low-frequency region.

## Figures and Tables

**Table 1:** Mass percentage (wt.%) of chemical components in GH3625 alloy.

Element	C	Si	Mn	Cr	Fe	Mo	Cu	W	Nb	Al	Ti	Ni
**Content**	0.04	0.20	0.20	21.0	4.40	8.00	0.04	0.08	3.60	0.30	0.20	61.7

**Table 2:** Segregation coefficient (k) of Nb in GH3625 (mean ± SD, n = 5).

Withdrawal Rate (μm/s)	Average Concentration of Dendrite Stem (wt%)	Average Interdendritic Concentration (wt%)	Average Segregation Ratio	Number of Data Points (Dendrite Trunk/ Interdendritic)
10	1.52 ± 0.13	6.01 ± 0.13	3.95	5/5
20	1. 18 ± 0.11015	1.71 ± 0.11015	1.45	5/5
50	1.79 ± 0.11	1.25 ± 0.11	1.43	5/5
100	1.37 ± 0.09074	1.47 ± 0.09074	1.07	5/5
200	1.36 ± 0.09074	1.55 ± 0.09074	1.14	5/5

**Table 3:** Segregation coefficients (k) of Mo in GH3625 (mean ± SD, n = 5).

Withdrawal Rate (μm/s)	Average Concentration of Dendrite Stem (wt%)	Average Interdendritic Concentration (wt%)	Average Segregation Ratio	Number of Data Points (Dendrite Trunk/ Interdendritic)
10	6.72 ± 0.13577	10.54 ± 0.13577	1.57	5/5
20	6.15 ± 0.13503	7.57 ± 0.13503	1.23	5/5
50	6.47 ± 0.11015	7.32 ± 0.11015	1.13	5/5
100	6.94 ± 0.13327	6.95 ± 0.13327	1.001	5/5
200	6.5 ± 0.09052	6.41 ± 0.09052	1.014	5/5

**Table 4:** Atomic percentage (at.%) of Laves content in the compound.

Element	Ni	Cr	Fe	Nb	Mo	C	Ti	Si
**Content (at.%)**	30–45%	10–16%	0–3%	4–16%	5–12%	0–3%	0–2%	0–3%

**Table 5:** Tensile properties of GH3625 alloy at different withdrawal rates (n = 3).

Withdrawal Rate (μm/s)	Ultimate Tensile Strength (MPa)	Yield Strength (MPa)
10	541	282
20	590	320
50	640	360
100	712	409
200	586	350

**Table 6:** Fitting results of potentiodynamic polarization curves (n = 3).

Processing Parameters	Withdrawal Rate (μm/s)	E_corr_/V	I_coor_/(A/cm^2^)
As-cast	10	−0.4288	1.166 × 10^−4^
	20	−0.23600	1.071 × 10^−4^
	50	−0.22067	9.801 × 10^−5^
	100	−0.21883	6.698 × 10^−5^
	200	−0.23488	1.315 × 10^−4^

## Data Availability

The original contributions presented in the study are included in the article. Further inquiries can be directed to the corresponding author.

## References

[1] Gao H., Zhang B., Fan Y., Zhijuan Z., Nan H., Tianli Z., Lei Z., Cai J., Wang K. (2024). Study on the Hot Deformation Behavior and Microstructure Evolution of As-Forged Gh3625 Alloy. J. Mater. Res. Technol..

[2] Reed R.C. (2008). The Superalloys: Fundamentals and Applications.

[3] Condruz M.R., Badea T.A., Paraschiv A. (2024). Compressive Behavior of Inconel 625 and Ti-6Al-4V Strut Lattices Fabricated by LPBF. Appl. Sci..

[4] Xu K., Cao J., Zheng Z., Zhao R., Xu G., Wang H., Wang J., Hur B., Yue X. (2025). Deformation Behavior of Inconel 625 Alloy with TPMS Structure. Materials.

[5] Gola K., Ledwig P., Dubiel B. (2023). Effect of microstructure of additively manufactured Inconel 625 on long-term corrosion behaviour in sulfuric acid media. JOM.

[6] Tang Q., Lin X., Zhao S., Niu Y., Sun H., Dong Y. (2020). Grain morphology and orientation effect on the high-temperature tensile behavior of directionally solidified magnesium alloy. Met. Mater. Int..

[7] Szeliga D., Kubiak K., Motyka M., Sieniawski J. (2016). Directional solidification of Ni-based superalloy castings: Thermal analysis. Vacuum.

[8] Peng P., Lu L., Liu Z., Xu Y., Zhang X., Ma Z., Zhang H., Guo M., Liu L. (2022). Investigation on influence ofTa on microstructure evolution of directionally solidified Ni-based superalloys. J. Alloys Compd..

[9] Yang Y., Wen Z., Pei H., Zhao Y., Yin X., Wang S., Yue Z. (2023). Effect of the withdrawal rate on the microstructure and creep behavior of the directionally solidified nickel-based superalloy. Prog. Nat. Sci. Mater. Int..

[10] Wang F., Wu Z., Ma D., Bührig-Polaczek A. (2017). Effect of directional solidification variables on the microstructures of single-crystal turbine blades of nickel-based superalloy. Adv. Eng. Mater..

[11] Yu J., Du D., Ren Z., Fautrelle Y., Moreau R., Li X. (2017). Influence of an axial magnetic field on microstructures and alignment in directionally solidified Ni-based superalloy. ISIJ Int..

[12] Li Q., Wang X., Zhao L., Xu L., Han Y. (2023). Validation and improvement in metallic material tensile models for small punch tests. J. Mater. Sci..

[13] Yang Z., Hu S., Chen Y., He M., Li W., Bai W., Wang X. (2025). Effect of Growth Orientation on Solidification Process of Nickel-Based Single Crystal Superalloy DD6. Metall. Mater. Trans. A.

[14] Alexandrov D.V., Galenko P.K. (2024). The Mullins–Sekerka theory: 60 years of morphological stability. J. Appl. Phys..

[15] Yan X., Xu Q., Tian G., Liu Q., Hou J., Liu B. (2021). Multi-scale modeling of liquid-metal cooling directional solidification and solidification behavior of nickel-based superalloy casting. J. Mater. Sci. Technol..

[16] Kavousi S., Zaeem M.A. (2025). Integration of multiscale simulations and machine learning for predicting dendritic microstructures in solidification of alloys. Acta Mater..

[17] Chen Y., Lv S.-M., Xie X.-F., Wen X.-C., Qu J.-L., Du J.-H. (2024). Solidification behaviour and hot cracking susceptibility of a novel Ni-based superalloy. J. Iron Steel Res. Int..

[18] Kong Y.F., Luo X.H., Li Y., Liu S. (2023). Effect of Gravity on Dendrite Growth and Microsegregation of Ni-based Single Crystal Superalloy. Chin. J. Mater. Res..

[19] Ding Y., Li H., Wang W., Liu J., Guo T., Hu Y. (2016). Microstructural Segregation and Homogenization Heat Treatment of Cast GH3625 Alloy Ingots. Mater. Sci. Eng..

[20] Fu K. (2025). Study on Microstructural Transformation and Molten Salt Corrosion Behavior of Inconel 625 Under High-Temperature Conditions Following High-Speed Laser Cladding. Ph.D. Thesis.

[21] Peng L., Takizawa S., Ikeda K.-I., Horiuchi T., Miura S. (2019). Effect of Si on the stability of NbCr2 Laves phase in Cr-Mo-Nb system. Intermetallics.

[22] Zhang Y., Gong W., Wang P., Li X. (2024). In Situ Observation of Microstructure and Precipitate Phase Transformation during the Solidification of Mg-Containing GH3625 Alloy at Different Cooling Rates. Steel Res. Int..

[23] Qin L., Ren P., Yi Y., Chen D., Lu Y., Sun D., Shi C., Zhou S. (2024). Microstructure, high-temperature cyclic oxidation, and hot corrosion behaviors of Inconel 718 alloy produced by laser-induction hybrid cladding. J. Mater. Res. Technol..

[24] Sun Y., Liu Y., Han J., Zhu Z., Zheng M., Song B., Chen W. (2024). Influence of enhanced Laves phase shape and distribution on atomic-scale frictional wear mechanisms in nickel-based single crystal alloys. Phys. Scr..

[25] Mishra R., Pandit D., Imam M. (2024). Microstructure, mechanical and corrosion study of friction stir processed Inconel 625 additive layers deposited via wire arc direct energy deposition. Addit. Manuf..

